# Construction of PLGA/JNK3-shRNA nanoparticles and their protective role in hippocampal neuron apoptosis induced by oxygen and glucose deprivation

**DOI:** 10.1039/c8ra00679b

**Published:** 2018-06-01

**Authors:** Jin Zheng, Jianguo Qi, Quan Zou, Zhenzhong Zhang

**Affiliations:** Department of Neurology, Tongde Hospital of Zhejiang Province Hangzhou 310012 Zhejiang Province China zhengjin22163@163.com zhangzhenzhong1023@163.com; Department of Neurology, Traditional Chinese Medical Hospital Affiliated to Xinjiang Medical University 830000 Urumqi Xinjiang Uygur Autonomous Region China qjg196623@sina.com; Department of Neurology, Wuxi Hospital of Traditional Chinese Medicine Wuxi 214000 Jiangsu Province China zouquan2663@163.com

## Abstract

C-Jun N-terminal kinase 3 (JNK3) activation plays an essential role in the pathophysiology of cerebral ischemia. However, to date, no specific interventions with good efficacy have been reported. Therefore, in this study, we constructed a PLGA/JNK3-shRNA nanoparticle and examined its effects on neuronal apoptosis in an *in vitro* model of cerebral ischemia (oxygen and glucose deprivation model, OGD model). Herein, three JNK3-specific siRNAs were designed and synthesized, and their effects on JNK mRNA transcription were investigated; the most efficacious JNK3-specific siRNA was selected for recombination of the GV107/JNK3-shRNA plasmid. The PLGA/JNK3-shRNA nanoparticle was constructed, and its surface characterizations were confirmed. The roles of PLGA/JNK3-shRNA in neuronal JNK3 mRNA transcription, protein expression and activation as well as cell apoptosis were examined in a rat hippocampal neuron OGD model and compared with those of Lipofectamine 2000-mediated JNK3-siRNA transfection. The recombinant plasmid GV107/JNK3-shRNA was successfully constructed using siRNA1928. The PLGA/JNK3-shRNA nanoparticles were prepared as a sphere with a complete shape and smooth surface. The particle was about 225.4 nm in diameter with an average drug loading of 36.9%. OGD can cause marked cell apoptosis, whereas PLGA/JNK3-shRNA exposure can partly inhibit apoptosis. Further analysis demonstrated that the levels of JNK3 mRNA and protein as well as their activation were suppressed by PLGA/JNK3-shRNA nanoparticles. Compared with JNK3-siRNA delivered by Lipofectamine-2000, PLGA/JNK3-shRNA nanoparticles induced more JNK3 mRNA and protein reduction and more anti-apoptotic effects. To conclude, the PLGA/JNK3-shRNA nanoparticles could achieve good effects on inhibiting JNK3 signaling and neuronal apoptosis, and their preparation was feasible.

## Abstract 2

C-Jun N-terminal kinase 3 (JNK3) activation plays an essential role in the pathophysiology of cerebral ischemia.

## Graphical abstract

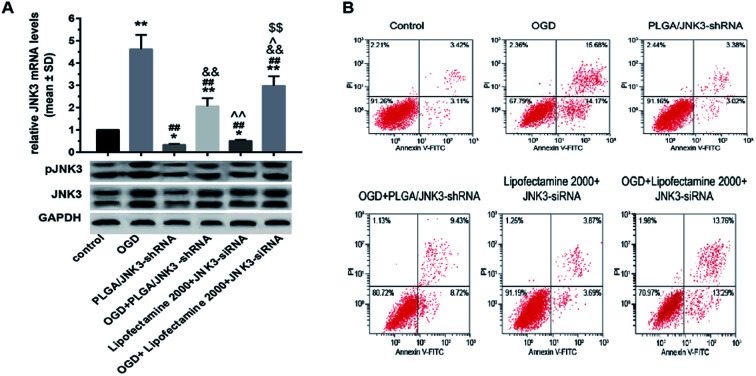

## 1. Introduction

Every year about 4 600 000 patients die of cerebral stroke, of which ischemic stroke accounts for about 85% deaths.^1^ Furthermore, 90% of stroke patients who survived were left with functional impairments.^2^ With the increasing number of elderly people, the risk of ischemic stroke is increasing and the related costs will impose a heavy burden on health system.^3^ However, the currently available therapies, such as thrombolysis, neurogenesis as well as angiogenesis, are far from satisfactory due to their limited effects and serious adverse effects.^4^ New and specific agents with less adverse effects for treating ischemic stroke have been anticipated in this context.

C-Jun N-terminal kinase (JNK) is a member of the family of serine and threonine mitogen-activated protein kinases (MAPK). There are three genes (JNK1, JNK2 and JNK3) that encode JNKs in human. Compared to JNK1 and JNK2, JNK3 is highly enriched in brain neurons. A number of studies have confirmed that JNK3 plays a key role in neuronal apoptosis,^5,6^ a typical cell pathophysiology associated with ischemic stroke.^4^ During the pathogenesis of cerebral ischemia, phosphorylation of JNK3 is enhanced, which then activates several pro-apoptotic genes *via* nuclear or non-nuclear pathways and eventually leads to neuronal apoptosis.^7,8^ Moreover, gene knock-out studies found that JNK3-deficient mice showed protection against ischemia and reduced ischemia-induced apoptosis as compared to the control.^9,10^ These studies suggest that JNK3 may become a new therapeutic target for treating ischemic stroke. Indeed, dozens of JNK inhibitors have been developed, and some have been tested pre-clinically for the treatment of cerebral ischemia.^11,12^ However, none of the drugs entered clinical trials due to their limited therapeutic efficacy or serious adverse effects. Additionally, these drugs encountered difficulties in crossing the blood-brain barrier (BBB).^13^

The development of RNA interference technology as well as vectors made it more possible to improve the efficacy of ischemic stroke treatment.^14,15^ Both viral and non-viral vectors have been tried. Although a non-viral vector-based drug delivery system usually shows lower efficiency than a viral vector-based drug delivery system, its preparation is simpler with higher loading capacity, lower cost and more safety.

Furthermore, by modifying the drugs with specific tags, non-viral vector-based delivery can achieve targeted transport.^16^ Among these non-viral vectors, poly lactic-*co*-glycolic acid (PLGA) has been widely used and certified by FDA as a medical material.^17^ In the present study, we constructed PLGA-based JNK3-shRNA nanoparticles and investigated their roles in JNK3 activity and hippocampal neuron apoptosis caused by OGD.

## 2. Materials and methods

### 2.1 Design and synthesis of JNK3 siRNAs

Herein, three JNK3-specific siRNAs, designated as siRNA1928 (si1928), si1929 and si1930, were designed based on the rat-derived JNK3 gene sequence (Accession no. DQ377224) using an online design software (Promega, USA). The individual sequences are shown in Table 1. The TTCAAGAGA sequence was designed in the loop structure to avoid termination, and splicing sites for BamHI and HindIII were designed at the 5′ end. All the designed sequences were aligned in the BLAST and synthesized by GenPharm Pharmaceutical Technology Co., Ltd (Shanghai).

**Table 1:** Sequences of siRNAs for the rat JNK3 gene

siRNA	Sense	Anti-sense
si1928	5′-CCAGUAACAUCGUAGUCAATT-3′	5′-UUGACUACGAUGUUACUGGTT-3′
si1929	5′-CCGAGCACAAUAAACUUAATT-3′	5′-UUAAGUUUAUUGUGCUCGGTT-3′
si1930	5′-GGAAAGAACUCAUCUACAATT-3′	5′-UUGUAGAUGAGUUCUUUCCTT-3′

### 2.2 Hippocampal neuronal cell culture and siRNA transfection

SD rat-derived hippocampal neuronal cells were purchased from Shanghai Jimian Biotechnology Co., Ltd and seeded in a 6-well cell culture plate at a density of 5 × 10^5^ cells per well. The cells were cultured in the NB medium containing 10% fetal bovine serum, 100 μg mL^−1^ penicillin and 100 g mL^−1^ streptomycin (Shanghai Yu duo of Health Science and Technology Co., Ltd.). Before siRNA transfection, the neurons were first placed in an incubator containing 5% CO_2_ at 37 °C for 24 h. Transfection was completed using Lipofectamine 2000 (Invitrogen, USA) following the manufacturer's instructions. Briefly, 1 μg of si1928, si1929 or si1930 was first dissolved in 50 μL of serum-free NB medium (solution A) and incubated at room temperature (RT) for 5 min. Then, 3 μL of Lipofectamine 2000 was added to 50 μL of a serum-free NB medium (solution B) and incubated at RT for 5 min. The two solutions were mixed together and incubated at RT for 30 min. Finally, the mixture was added to the cells dropwise followed by incubation under an atmosphere of CO_2_. After 48 h incubation, the cells were obtained for transfection efficacy identification.

### 2.3 Total RNA extraction and real-time PCR

Cells were lysed with 1 mL of Trizol at RT for 5 min followed by the addition of 0.2 mL of chloroform. After incubation, the mixture was centrifuged at 10 000 g for 15 min. Then, the upper aqueous phase was transferred into a new EP tube and mixed with 0.5 mL of isopropyl alcohol. After 10 min of incubation, the mixture was centrifuged to obtain RNA precipitates, which were washed, dried and dissolved in an RNase-free water. A total of 2 μg of RNA was used to synthesize cDNA using the PrimeScript RT Reagent Kit (Takara, JPN). Real-time PCR was performed using a SYBR premix ex Taq™ Kit (Roche, USA) for JNK3 mRNA. β-Actin was amplified as an endogenous control. The primer sequences for the amplification of JNK3 and β-actin were as follows: JNK3, sense: 5′-CTG ATG CAG TGC ACG ATC TAC-3′, anti-sense: 5′-AGC GTC GTA CTA GAC GTT GCG AT-3′^18^ and β-actin, sense: 5′-GTAGCCATCCAGGCTGTGTT-3′, anti-sense: 5′-CCCTCATAGATGGGCACAGT-3′.^19^ The relative levels of JNK3 mRNA were calculated using the comparative *C*_t_ method formula: 2^−Δ*C*_t_^.

### 2.4 Construction and identification of GV107/JNK3-shRNA

**Fig. 1:**
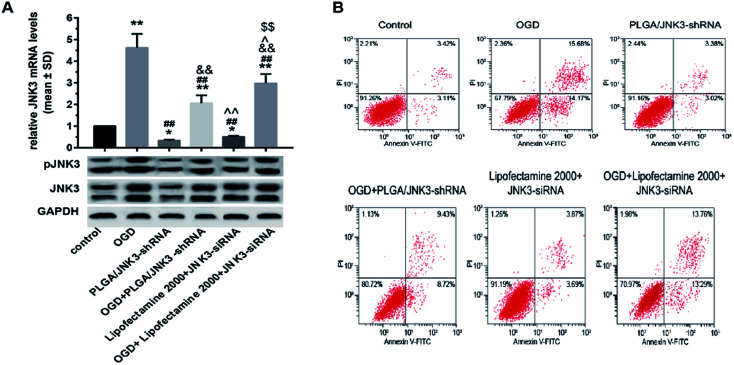
The efficient inhibition of OGD-induced JNK3 expression and the subsequent neuronal apoptosis by PLGA/JNK3-shRNA nanoparticles.

The most powerful siRNA for JNK3 mRNA suppression was chosen for constructing GV107/JNK3-shRNA. At first, 10 μM of shRNA was obtained from single-stranded oligo siRNA after annealing. The annealing process was set as follows: 95 °C for 5 min, 85 °C for 5 min, 75 °C for 5 min and 70 °C for 5 min. Then, the synthesized shRNA and GV107 vector containing human H1 promoter, GFP gene and Neo resistant gene (Genpharma, Shanghai) were subjected to digestion with both BamHI and HindIII. After this, the digested products were ligated at 16 °C overnight and transformed into the competent cells DH5a (Takara Co., Ltd.). The cells were streaked onto a LB plate containing ampicillin and incubated at 37 °C overnight for selection. Finally, the plasmid was extracted and identified by enzyme digestion. Fig. 1 shows a schematic of the plasmid GV107.

### 2.5 Preparation of PLGA/JNK3-shRNA nanoparticles

PLGA/JNK3-shRNA nanoparticles were prepared by a multiple emulsion method. Briefly, 40 mg of PLGA (sigma, USA) was dissolved in 10 mL of dichloromethane to serve as the organic phase, 15 mg polyvinyl alcohol (TCI, USA) was dissolved in 20 mL of distilled water to serve as the external water phase, and JNK3-shRNA plasmids were added to 3 mL of distilled water to serve as the internal water phase. The internal water phase was slowly added to the organic phase to develop a W/O primary emulsion using a magnetic stirrer. Then, the external water phase was added to the W/O primary emulsion to form a W/O/W multiple emulsion using an ultrasonic probe (JY92-IIN, Ningbo Xingyi Ultrasonic Equipment Co., Ltd.). Finally, the organic solvent dichloromethane was evaporated to obtain the lyophilized PLGA/JNK3-shRNA nanoparticles.

### 2.6 Characteristic analysis of PLGA/JNK3-shRNA nanoparticles

The size of the nanoparticles was measured using a laser particle size analyzer (Mastersizer 2000, Malvern Instruments Ltd, UK). Briefly, the lyophilized nanoparticles were immersed in deionized water for ultrasonic dispersion (CQ-600, Shanghai Shenxi Ultrasonic Instrument Co., Ltd.). After dispersion, the liquid samples were either added to a dish to analyze the size or dropped on the surface of a dish to dry up naturally for observing the surface structure using a scanning electron microscope.

Drug loading was analyzed and calculated as follows: 2.5 mg of lyophilized nanoparticles was placed in a 1.5 mL EP tube, and then, 250 μL of TE and 1 mL of chloroform were added to the tube sequentially to dissolve the nanoparticles. After this, the solution was incubated using a shaker (200 rpm) at RT for 60 min. After centrifugation at 12 000 rpm for 5 min, the supernatant was obtained and used for DNA quantification. The amount of the plasmid was calculated using the following formula: drug loading (%) = the amount of encapsulated plasmid/total nanoparticles.

### 2.7 Oxygen and glucose deprivation (OGD) model and experimental design

We further tested the effects of PLGA/JNK3-shRNA nanoparticles on neuronal JNK activation and apoptosis caused by OGD, a widely used *in vitro* model of cerebral ischemia.^20^ Rat hippocampal neuronal cells were divided into six groups in this model: control (no treatment); OGD (OGD process only); PLGA/JNK3-shRNA (PLGA/JNK3-shRNA nanoparticles exposure only); OGD + PLGA/JNK3-shRNA; Lipofectamine 2000 + JNK3-siRNA; and OGD + Lipofectamine 2000 + JNK3-siRNA.

The OGD model was achieved using the following procedures. Rat hippocampal neuronal cells (5 × 10^5^ per well) were seeded into a 6-well plate and incubated under an atmosphere of 5% CO_2_ at 37 °C for 24 h. Then, the cells were cultured in a serum-free and sugar-free NB medium and placed in a tri-gas incubator with low oxygen and high nitrogen contents (2% O_2_, 5% CO_2_, and 93% N_2_) for 48 hours.^21^ The control cells were incubated under normoxic conditions for the same time period. After inducing hypoxia, the cells were subjected to further analyses.

To evaluate the transfection and gene silencing efficiency, JNK siRNAs were delivered through PLGA/JNK3-shRNA nanoparticles or Lipofectamine 2000. The PLGA/JNK3-shRNA nanoparticles were added to the culture medium at a final concentration of 100 g mL^−1^ after incubation under hypoxia for 6 hours. JNK siRNA (0.8 μg siRNA per well) was transfected using Lipofectamine 2000 (5 μL) into the cells for 6 hours.^22^ After transfection, the medium was replaced with a fresh culture medium followed by incubation.

### 2.8 Morphological detection of apoptosis by Hoechst 33258 staining

Hoechst 33258 staining was used for morphological detection of apoptosis according to the protocols described by Hao *et al.*^23^ and Kasibhatla *et al.*^24^ After inducing hypoxia, the cells in each group were washed once with PBS and fixed in 4% polyformalin (PFA) at RT for 30 min. After washing three times with PBS, the cells were stained with the Hoechst 33258 staining solution at RT for 10 min. The cells were observed and imaged using a fluorescence microscope (Olympus CKX41, Japan), and 10 fields were selected randomly for counting.

### 2.9 Flow cytometry (FCM) analysis of apoptosis by FITC-Annexin V/PI double staining

We further detected cell apoptosis using the Annexin-V/PI double-staining method *via* flow cytometry.^18^ Briefly, after treatment, the cells were washed with cold PBS, centrifuged twice at 2000 rpm for 5 min and then resuspended in a binding buffer. After this, the suspension was mixed with 5 μL of APC and 5 μL of PI and then loaded onto a FACSCalibur flow cytometer (BD; San Jose, CA, USA) for detection. Annexin V^+^/PI^−^ presents early apoptotic cells, and Annexin V^+^/PI^+^ presents late apoptotic cells. The neuronal apoptotic ratio was calculated by including both Annexin V^+^/PI^−^ and Annexin V^+^/PI^+^ cells.

### 2.10 Protein extraction and western-blot

Cells in each group were obtained in an Eppendorf tube containing 400 μL of pre-cooled RIPA lysates (Beyotime, China) followed by incubation on ice for 30 min and then centrifugation at 12 000 rpm at 4 °C for 10 min. The supernatant was obtained and quantified by the BCA method. The protein concentration was adjusted to 5 μg μL^−1^ before use. Herein, 25 μg of protein was loaded on 10% polyacrylamide gel. After SDS-PAGE, the protein was transferred onto a PVDF membrane (100 V, 1 h). The membrane was incubated in 5% BSA at 37 °C for 60 min and then incubated with a goat anti-rabbit JNK3 antibody (1 : 1000, ab126591, Abcam, USA) or pJNK3 antibody (1 : 1000, ab124956) overnight at 4 °C; GAPDH was used as an endogenous control. After final washing with PBS, the membrane was incubated with a HRP-conjugated secondary antibody (1 : 10 000) at RT for 1 h. The blots were visualized using an enhanced chemiluminescence reaction (ECL) system, and densitometry analysis was performed using the Quantity One software (Bio-Rad).

### 2.11 Statistical analysis

SPSS 19.0 was used for statistical analysis. All data were presented as mean ± SD. One-way ANOVA with *post hoc* Tukey tests was used to analyze the differences in mRNA and protein levels or cell counts among groups. A *P* value of less than 0.05 (two-tailed) was considered to be statistically significant.

## 3. Results

### 3.1 Si1928 as the most powerful siRNA to inhibit JNK3 mRNA transcription

**Fig. 2:**
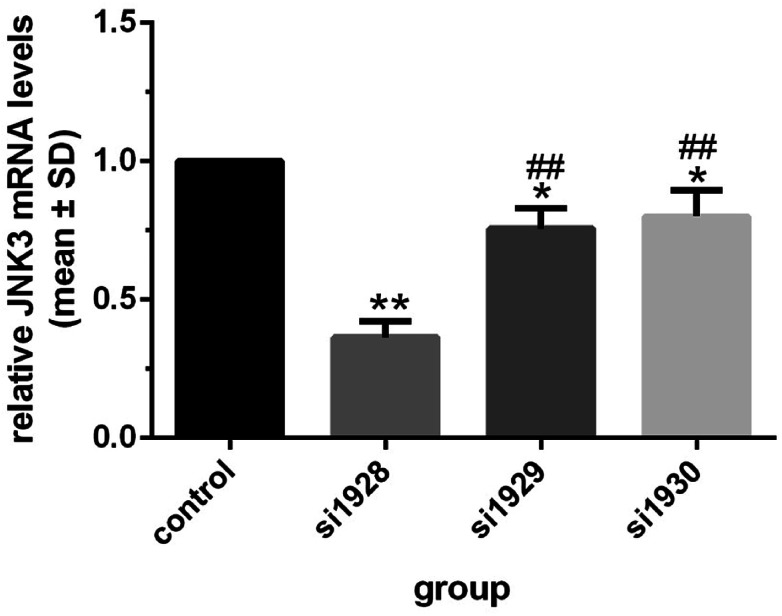
Hippocampal neuron JNK3 mRNA levels after JNK3 siRNA transfection *P < 0.05 and **P < 0.01 *vs.* control; ^##^*P* < 0.01 *vs.* si1928. Data were obtained from three independent experiments.

The inhibitory effects of three predesigned JNK3-specific siRNAs on JNK3 mRNA transcription were tested by transient transfection of siRNAs into SD rat hippocampal neuronal cells. As shown in Fig. 2, compared with other groups, si1928 provided the most powerful inhibition of neuronal JNK3 mRNA levels and was chosen to construct the GV107/JNK3-shRNA recombinant plasmid.

### 3.2 Successful construction of GV107/JNK3-shRNA

**Fig. 3:**
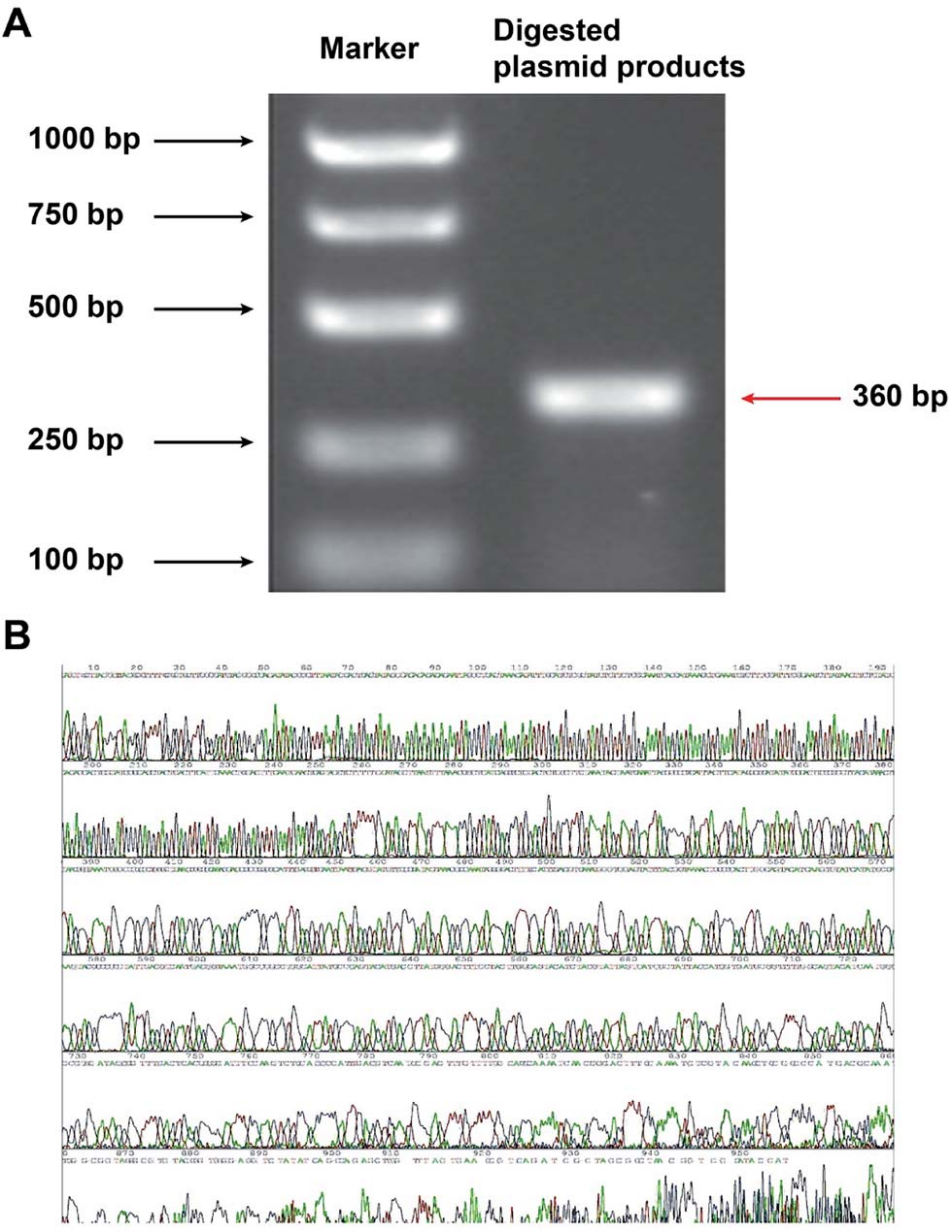
Confirmation of the construction of the GV107/JNK3-shRNA recombinant plasmid by electrophoresis. (A) The constructed plasmid was spliced by BamHI and HindIII, and the digested products were subjected to electrophoresis. (B) Sequencing results of the GV107/JNK3-shRNA recombinant plasmid.

The constructed plasmid was subjected to dual digestion with BamHI and HindIII, and the length of the target fragment was confirmed by electrophoresis. As shown in Fig. 3A, the obtained fragment had a length of 360 bps, which was in accordance with the expected size of 366 bps. The sequencing result further confirmed the correct construction of the GV107/JNK3-shRNA recombinant plasmid (Fig. 3B).

### 3.3 Characteristics of the PLGA/JNK3-shRNA nanoparticles

**Fig. 4:**
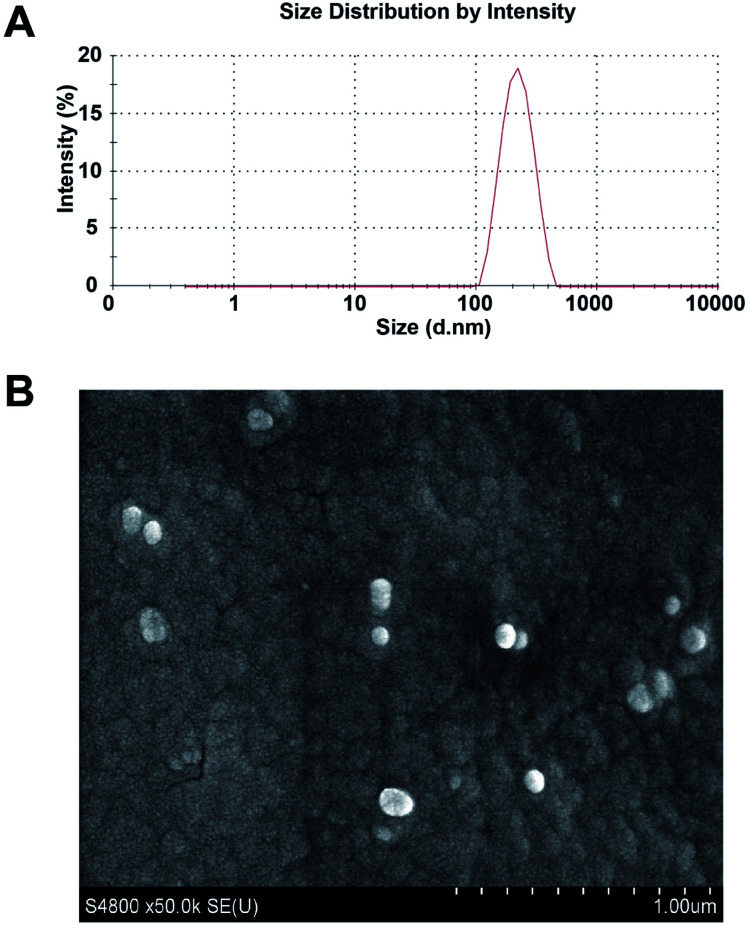
Characteristics of the PLGA/JNK3-shRNA nanoparticles. (A) Size distribution patterns of the constructed particles. (B) Surface morphology of the nanoparticles detected by scanning electron microscopy.

Fig. 4A displays the diameter of the constructed particles detected using a laser particle size analyzer. The average diameter of the nanoparticles was 225.4 nm with a polydispersity index of 0.098. The drug loading of PLGA/JNK3-shRNA nanoparticles could reach 36.9%. Scanning electron microscopy showed that the PLGA/JNK3-shRNA nanoparticles were spherical, with a complete shape and smooth surface (Fig. 4B).

### 3.4 PLGA/JNK3-shRNA nanoparticles efficiently inhibited neuronal JNK3 expression and apoptosis induced by OGD

**Fig. 5:**
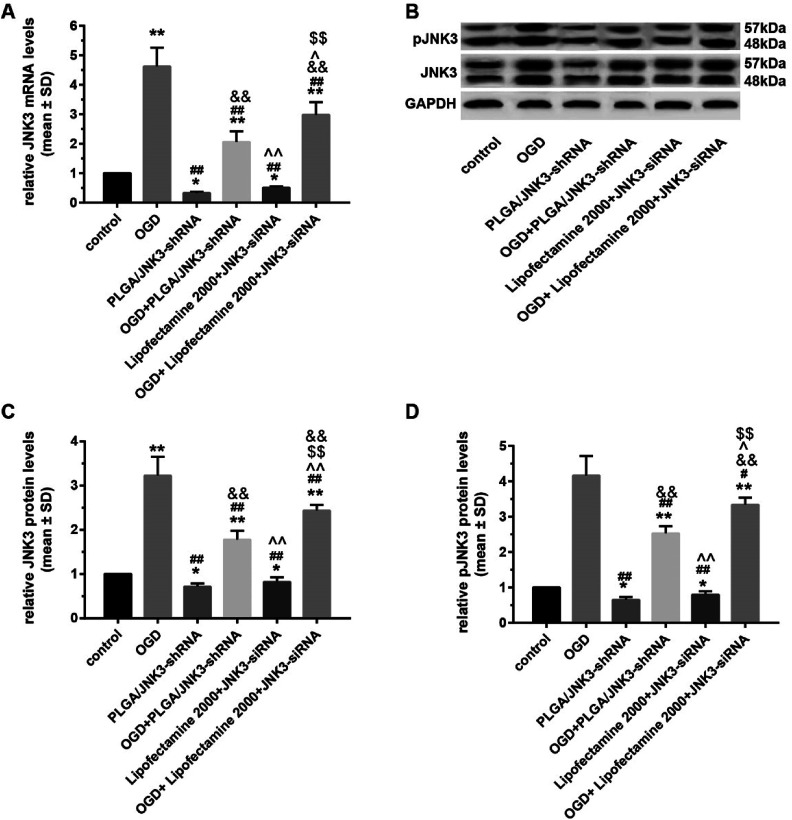
Effects of PLGA/JNK3-shRNA nanoparticles on OGD-induced JNK3 mRNA transcription and protein translation. (A) More efficient inhibition of OGD-induced JNK3 mRNA transcription by PLGA/JNK3-shRNA nanoparticles than by Lipofectamine-based JNK3 siRNA transfection. (B) Representative blot images among groups for JNK3 protein expression and phosphorylation. (C) More efficient inhibition of JNK3 protein expression after OGD by nanoparticles than by Lipofectamine-based JNK3 siRNA transfection. (D) JNK3 phosphorylation was more suppressed by nanoparticles than by Lipofectamine-based JNK3 siRNA transfection. Group: control = no treatment; OGD = oxygen and glucose deprivation; PLGA/JNK3-shRNA = PLGA/JNK3-shRNA nanoparticle treatment; OGD + PLGA/JNK3-shRNA = PLGA/JNK3-shRNA nanoparticle treatment after incubation under hypoxia for 6 h; Lipofectamine 2000 + JNK3 siRNA = Lipofectamine 2000-based JNK3 siRNA transfection for 6 h; OGD + Lipofectamine 2000 + JNK3 siRNA = Lipofectamine 2000-based JNK3 siRNA transfection for 6 h after incubation under hypoxia. *P < 0.05 and **P < 0.01 *vs.* control; ^#^*P* < 0.05 and ^##^*P* < 0.01 *vs.* OGD; ^&&^*P* < 0.01 *vs.* PLGA/JNK3-shRNA; ^^^*P* < 0.05 and ^^^^*P* < 0.01 *vs.* OGD + PLGA/JNK3-shRNA; ^$$^*P* < 0.01 *vs.* Lipofectamine 2000 + JNK3 siRNA. Data were obtained from three independent experiments.

Transcriptional and translational levels of JNK3 in hippocampal neurons subjected to OGD were detected to confirm the effects of PLGA/JNK3-shRNA nanoparticles. As shown in Fig. 5A, after OGD, neurons exhibited significant elevation of JNK3 mRNA levels. The addition of PLGA/JNK3-shRNA nanoparticles to the culture medium caused a significant reduction in JNK3 mRNA levels as compared to the case of the OGD group. Furthermore, the inhibitory efficiency was more powerful when compared with that of JNK3 siRNA delivered by Lipofectamine 2000. Compared with the case of the OGD + Lipofectamine 2000 + JNK3 siRNA group, JNK3 mRNA levels were significantly lower in cells treated with PLGA/JNK3-shRNA nanoparticles.

With respect to the protein levels of JNK3, the results indicate that OGD can induce significant elevation of JNK3 expression in comparison with the control (Fig. 5B and C). The PLGA/JNK3-shRNA nanoparticles alone had no effect on JNK3 expression and could partly reverse OGD-induced enhancement of JNK3 expression. Compared with those in the OGD + Lipofectamine 2000 + JNK3 siRNA group, the JNK3 protein levels were significantly lower in cells treated with PLGA/JNK3-shRNA nanoparticles.

With regard to the JNK3 phosphorylation levels, a similar effect was observed on JNK3 expression upon exposure to PLGA/JNK3-shRNA nanoparticles, and JNK3 siRNA delivered by Lipofectamine 2000 showed less JNK3 protein downregulation than that delivered by PLGA/JNK3-shRNA nanoparticles (Fig. 5B and D).

**Fig. 6:**
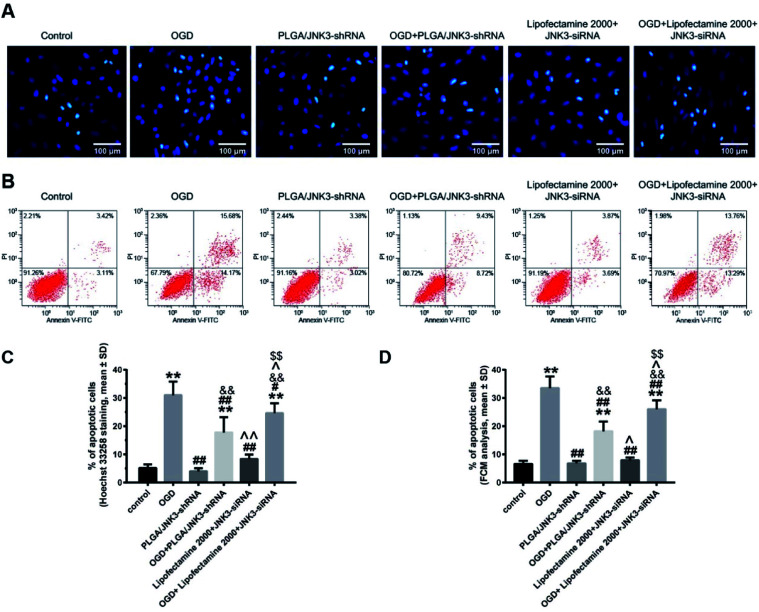
Effects of PLGA/JNK3-shRNA nanoparticles on the OGD-induced neuron apoptosis. (A) Representative Hoechst 33258 staining figures showing the effects of OGD and/or PLGA/JNK3-shRNA on hippocampal neuron apoptosis. (B) Representative Annexin V/PI flow cytometric assay results of neuronal apoptosis. (C) Histograms showing that the nanoparticles inhibited OGD-induced cell apoptosis, evaluated by Hoechst 33258 staining, more efficiently than Lipofectamine 2000-based JNK3 siRNA transfection. (D) Histograms showing that the nanoparticles inhibited OGD-induced cell apoptosis, evaluated by flow cytometry (FCM), more efficiently than Lipofectamine 2000-based JNK3 siRNA transfection. PLGA/JNK3-shRNA = PLGA/JNK3-shRNA nanoparticle treatment; OGD + PLGA/JNK3-shRNA = PLGA/JNK3-shRNA nanoparticle treatment after incubation under hypoxia for 6 h; Lipofectamine 2000 + JNK3 siRNA = Lipofectamine 2000-based JNK3 siRNA transfection for 6 h; OGD + Lipofectamine 2000 + JNK3 siRNA = Lipofectamine 2000-based JNK3 siRNA transfection for 6 h after incubation under hypoxia. **P < 0.01 *vs.* control; ^#^*P* < 0.05 and ^##^*P* < 0.01 *vs.* OGD; ^&&^*P* < 0.01 *vs.* PLGA/JNK3-shRNA; ^^^*P* < 0.05 and ^^^^*P* < 0.01 *vs.* OGD + PLGA/JNK3-shRNA; ^$$^*P* < 0.01 *vs.* Lipofectamine 2000 + JNK3 siRNA. Data were obtained from three independent experiments.

We then investigated the effects of inhibited JNK3 activation by PLGA/JNK3-shRNA nanoparticles on cell apoptosis by the Hoechst 33258 staining method and flow cytometry analysis. As shown in Fig. 6A, the nuclei in the control and siRNA groups were round or oval with a normal blue color, whereas the nuclear membrane was intact. After OGD processing, the nuclei shrunk and turned bright blue, indicating cellular apoptosis. Statistical analysis based on Hoechst 33258 staining indicated that OGD induced significant elevation of cell apoptosis rate when compared with the control, and pretreatment with PLGA/JNK3-shRNA nanoparticles could partly reverse the pro-apoptotic effects of OGD (Fig. 6C). Moreover, compared with OGD + Lipofectamine 2000 + JNK3 siRNA group, the OGD + PLGA/JNK3-shRNA nanoparticle group showed less neuron apoptosis. No independent effect was observed for PLGA/JNK3-shRNA nanoparticle exposure only. Lipofectamine 2000-based JNK3 siRNA transfection induced a slightly increased cell apoptosis when compared with the control, but the difference was not significant.

The anti-apoptotic effects of PLGA/JNK3-shRNA nanoparticle treatment were further verified by the flow cytometry analysis. As shown in Fig. 6B and D, OGD-induced neuron apoptosis could be significantly reduced by either PLGA/JNK3-shRNA nanoparticle treatment or Lipofectamine 2000-based JNK3 siRNA transfection, and less apoptosis was observed in cells treated with PLGA/JNK3-shRNA nanoparticles when compared with those subjected to Lipofectamine 2000-based JNK3 siRNA transfection. No significant effects on cell apoptosis were observed by either PLGA/JNK3-shRNA nanoparticle or Lipofectamine 2000/JNK3 siRNA treatment alone.

## 4. Discussion

In this study, we have successfully constructed PLGA/JNK3-shRNA nanoparticles using a pre-selected JNK3-specific siRNA. Functional studies using a hippocampal neuron OGD model, an *in vitro* manifestation of cerebral ischemia, suggested that the PLGA/JNK3-shRNA nanoparticles could efficiently inhibit OGD-induced JNK3 expression and the subsequent neuronal apoptosis.

Specifically expressed in central nervous system neurons, JNK3 has long been attempted as a target for treating ischemic stroke.^9^ Borsello *et al.* first designed a cell-penetrating JNK inhibitor-D-JNKI1 and confirmed its powerful protection against neuronal hypoxia/ischemia.^12,25^ However, D-JNKI1 was not JNK3 specific. It was harmful at high doses and was neuroprotective only when excitotoxicity-induced endocytosis was working.^26^ These shortcomings hindered the clinical application of D-JNKI1. Other JNK inhibitors under test were also limited by non-specificity, inferiority in crossing the blood-brain barrier or adverse effects.^11,27^ Under these circumstances, specific post-transcriptional silencing of JNK3 using siRNA has been a promising strategy.^28,29^ However, siRNA therapy has its own limitations such as rapid degradation, poor cellular uptake, non-specific distribution and low endosomal escape efficiency.^30^ To overcome these shortcomings, polyelectrolyte complexes made with cationic polymers, peptides, or lipids have been tried.^30^ These complexes helped to protect siRNA from degradation and facilitated cellular uptake of siRNA, but also raised concerns about cytotoxicity and stability.^31,32^ Therefore, efficient carrier materials that are non-toxic, biocompatible and biodegradable are still needed.

As a novel drug nanocarrier, PLGA is gaining attention in controlled delivery of specific drugs.^17^ PLGA has been approved by the US FDA for use in drug delivery systems due to its controlled and sustained-release properties, low toxicity, and biocompatibility with tissue and cells.^33^ A variety of small molecular drugs, such as paclitaxel, cisplatin and curcumin, were successfully carried by PLGA, and they produced the anticipated effects.^34,35^ Furthermore, by proper surface modifications, the engineered PLGA nanoparticles could pass the BBB; this made PLGA one of the most promising drugs and gene delivery systems for crossing the BBB.^36,37^ Currently, there are no studies that specifically target brain JNK3 to achieve brain protection. Herein, we have designed and constructed the GV107/JNK3-shRNA recombinant plasmid and then prepared PGLA/JNK3-shRNA nanoparticles using a multiple emulsion method. We found that the constructed nanoparticle was a sphere with a diameter of 225.4 nm and smooth surface. The average drug loading rate was 36.9%. These results indicated that the PLGA/JNK3-shRNA nano-microspheres could be prepared easily with high drug loading, which might meet the clinical demands for treating ischemic stroke.

We further examined the potential of PLGA/JNK3-shRNA nanoparticles in treating cerebral ischemia using the *in vitro* OGD model. Relevant results confirmed that the engineered nanoparticles could efficiently suppress OGD-induced increase in JNK expression and activation as well as neuronal apoptosis. These findings were in line with those of previous studies that highlighted an effect of inhibition of JNK3 activation on the reduction of cell death during cerebral ischemia.^9,38,39^ Guan *et al.* reported Tat-JBD that hindered the assembly of JIP-1-JNK3 complexes, and the subsequent JNK3 activation was neuroprotective no matter whether Tat-JBD was used before or after hippocampal ischemia.^8^ This finding further supported the clinically practicability of neural-specific JNK3 inhibitors. Additionally, the results of this study indicate that PLGA can be used as a drug carrier with high performance. Previous studies have demonstrated that PLGA presents many advantages, such as avoiding the degradation by lysosomes or nuclease, as a drug carrier.^40,41^ It could improve the efficacy of gene expression in the cells. Moreover, PLGA is safe, non-toxic and degradable, and its long-term use will not lead to immune tolerance to the carrier.^36^

Our study found that PLGA/JNK3-shRNA nanoparticles were more efficient than Lipofectamine 2000-based JNK3 siRNA transfection for inhibiting JNK3 expression and the subsequent anti-apoptosis (Fig. 5 and 6). These results were similar to those of other studies that used similar PLGA-based nanoparticles.^22,30,42^ The explanations are as follows: first, the double emulsion solvent evaporation technique during the preparation of PLGA/JNK3-shRNA nanoparticles is a useful tool for encapsulation of hydrophilic molecules such as shRNA.^43^ Gomes *et al.* reported a 50% siRNA association efficiency by PLGA nanoparticles.^42^ Second, PLGA nanoparticles ensured better intracellular uptake. Mukerjee *et al.* reported that PLGA nanoparticle showed better intracellular uptake than Lipofectamine-shRNA complexes and substantially higher transfection efficiency.^30^ Third, lower toxicity of PLGA nanoparticles helped keep the cells viable. As is known, Lipofectamine has high toxicity and can cause cell apoptosis alone.^44^ In contrary, PLGA is highly biocompatible and seldom causes cell injury normally.^45^ However, we compared the transfection efficiency at only one time point. There was still a possibility that the transfection efficiency was different in the long term.

There are at least two caveats that should not be ignored in the present study. First, only one dose of PLGA/JNK3-shRNA nanoparticles was tried in our experiment, which made the extensive evaluation of therapeutic or toxic effects impossible. Second, we only tested the role of the constructed nanoparticles on neuronal apoptosis *in vitro*. No studies have been performed to evaluate the crossing ability and confirm the neuroprotective effects *in vivo*. Further animal studies were needed to validate the treating role of PLGA/JNK3-shRNA nanoparticles in ischemic stroke.

To conclude, the PLGA/JNK3-shRNA nanoparticles were successfully constructed and were effective in reducing hypoxia-induced neuronal apoptosis through specifically inhibiting JNK3 activation.

## Conflicts of interest

There are no conflicts to declare.

## Supplementary Material

## References

[cit1] Takenaka K., Kato M., Yamauti K., Hayashi K. (2014). J Stroke Cerebrovasc. Dis..

[cit2] Hinkle J. L., Guanci M. M. (2007). J. Neurosci. Nurs..

[cit3] Feigin V. L., Forouzanfar M. H., Krishnamurthi R., Mensah G. A., Connor M., Bennett D. A., Moran A. E., Sacco R. L., Anderson L., Truelsen T., O'Donnell M., Venketasubramanian N., Barker-Collo S., Lawes C. M., Wang W., Shinohara Y., Witt E., Ezzati M., Naghavi M., Murray C., I. Global Burden of Diseases, S. Risk Factors, G. B. D. S. E. G. (2014). Lancet.

[cit4] Gervois P., Wolfs E., Ratajczak J., Dillen Y., Vangansewinkel T., Hilkens P., Bronckaers A., Lambrichts I., Struys T. (2016). Med. Res. Rev..

[cit5] Resnick L., Fennell M. (2004). Drug Discovery Today.

[cit6] Xia Z., Dickens M., Raingeaud J., Davis R. J., Greenberg M. E. (1995). Science.

[cit7] Tian H., Zhang G., Li H., Zhang Q. (2003). Neurosci. Res..

[cit8] Guan Q. H., Pei D. S., Zong Y. Y., Xu T. L., Zhang G. Y. (2006). Neuroscience.

[cit9] Kuan C. Y., Whitmarsh A. J., Yang D. D., Liao G., Schloemer A. J., Dong C., Bao J., Banasiak K. J., Haddad G. G., Flavell R. A., Davis R. J., Rakic P. (2003). Proc. Natl. Acad. Sci. U. S. A..

[cit10] Pirianov G., Brywe K. G., Mallard C., Edwards A. D., Flavell R. A., Hagberg H., Mehmet H. (2007). J. Cereb. Blood Flow Metab..

[cit11] Gehringer M., Muth F., Koch P., Laufer S. A. (2015). Expert Opin. Ther. Pat..

[cit12] Hirt L., Badaut J., Thevenet J., Granziera C., Regli L., Maurer F., Bonny C., Bogousslavsky J. (2004). Stroke.

[cit13] Hersh D. S., Wadajkar A. S., Roberts N. B., Perez J. G., Connolly N. P., Frenkel V., Winkles J. A., Woodworth G. F., Kim A. J. (2016). Curr. Pharm. Des..

[cit14] Saraiva C., Praca C., Ferreira R., Santos T., Ferreira L., Bernardino L. (2016). J. Controlled Release.

[cit15] Sanchez-Purra M., Ramos V., Petrenko V. A., Torchilin V. P., Borros S. (2016). Int. J. Pharm..

[cit16] Kaur J., Tikoo K. (2015). Oncogene.

[cit17] Jain A. K., Das M., Swarnakar N. K., Jain S. (2011). Crit. Rev. Ther. Drug Carrier Syst..

[cit18] Liu G., Song J., Guo Y., Wang T., Zhou Z. (2013). Behav. Brain Funct..

[cit19] Kenchappa R. S., Tep C., Korade Z., Urra S., Bronfman F. C., Yoon S. O., Carter B. D. (2010). J. Biol. Chem..

[cit20] Thompson R. J., Zhou N., MacVicar B. A. (2006). Science.

[cit21] Vavilis T., Delivanoglou N., Aggelidou E., Stamoula E., Mellidis K., Kaidoglou A., Cheva A., Pourzitaki C., Chatzimeletiou K., Lazou A., Albani M., Kritis A. (2016). Cell. Mol. Neurobiol..

[cit22] Du J., Sun Y., Shi Q. S., Liu P. F., Zhu M. J., Wang C. H., Du L. F., Duan Y. R. (2012). Int. J. Mol. Sci..

[cit23] Hao L. N., Zhang Q. Z., Yu T. G., Cheng Y. N., Ji S. L. (2011). Brain Res..

[cit24] Kasibhatla S., Amarante-Mendes G. P., Finucane D., Brunner T., Bossy-Wetzel E., Green D. R. (2006). Cold Spring Harb. Protoc..

[cit25] Borsello T., Clarke P. G., Hirt L., Vercelli A., Repici M., Schorderet D. F., Bogousslavsky J., Bonny C. (2003). Nat. Med..

[cit26] Vaslin A., Naegele-Tollardo S., Puyal J., Clarke P. G. (2011). J. Neurochem..

[cit27] Yarza R., Vela S., Solas M., Ramirez M. J. (2015). Front. Pharmacol..

[cit28] Tonges L., Planchamp V., Koch J. C., Herdegen T., Bahr M., Lingor P. (2011). J. Mol. Neurosci..

[cit29] Ezanno H., Pawlowski V., Abdelli S., Boutry R., Gmyr V., Kerr-Conte J., Bonny C., Pattou F., Abderrahmani A. (2014). J. Diabetes Res..

[cit30] Mukerjee A., Shankardas J., Ranjan A. P., Vishwanatha J. K. (2011). Nanotechnology.

[cit31] Lee S. H., Bae K. H., Kim S. H., Lee K. R., Park T. G. (2008). Int. J. Pharm..

[cit32] Wang Y., Li S. Y., Shen S., Wang J. (2018). Biomaterials.

[cit33] Sadat Tabatabaei Mirakabad F., Nejati-Koshki K., Akbarzadeh A., Yamchi M. R., Milani M., Zarghami N., Zeighamian V., Rahimzadeh A., Alimohammadi S., Hanifehpour Y., Joo S. W. (2014). Asian Pac. J. Cancer Prev..

[cit34] Moreno D., Zalba S., Navarro I., Tros de Ilarduya C., Garrido M. J. (2010). Eur. J. Pharm. Biopharm..

[cit35] Yallapu M. M., Maher D. M., Sundram V., Bell M. C., Jaggi M., Chauhan S. C. (2010). J. Ovarian Res..

[cit36] Cai Q., Wang L., Deng G., Liu J., Chen Q., Chen Z. (2016). Am. J. Transl. Res..

[cit37] Nikandish N., Hosseinzadeh L., Hemati Azandaryani A., Derakhshandeh K. (2016). Iran. J. Pharm. Res..

[cit38] Zhu Q. J., Kong F. S., Xu H., Wang Y., Du C. P., Sun C. C., Liu Y., Li T., Hou X. Y. (2014). Proc. Natl. Acad. Sci. U. S. A..

[cit39] Pan J., Li H., Zhang B., Xiong R., Zhang Y., Kang W. Y., Chen W., Zhao Z. B., Chen S. D. (2015). PLoS One.

[cit40] Panyam J., Zhou W. Z., Prabha S., Sahoo S. K., Labhasetwar V. (2002). FASEB J..

[cit41] Wang H., Zhao P., Su W., Wang S., Liao Z., Niu R., Chang J. (2010). Biomaterials.

[cit42] Gomes M. J., Fernandes C., Martins S., Borges F., Sarmento B. (2017). Iran. J. Pharm. Res..

[cit43] Wang L., Griffel B., Xu X. (2017). Methods Mol. Biol..

[cit44] Rao S., Morales A. A., Pearse D. D. (2015). BioMed Res. Int..

[cit45] Grabowski N., Hillaireau H., Vergnaud J., Santiago L. A., Kerdine-Romer S., Pallardy M., Tsapis N., Fattal E. (2013). Int. J. Pharm..

